# Prävalenz der Hypophosphatasie bei adulten Patienten in der Rheumatologie

**DOI:** 10.1007/s00393-021-00994-5

**Published:** 2021-04-14

**Authors:** P. Karakostas, R. Dolscheid-Pommerich, M. D. Hass, N. Weber, P. Brossart, V. S. Schäfer

**Affiliations:** 1 Klinik für Innere Medizin III, Onkologie, Hämatologie, Rheumatologie und Klinische Immunologie, Universitätsklinikum Bonn, Venusberg Campus 1, 53127 Bonn, Deutschland; 2 Institut für Klinische Chemie und Klinische Pharmakologie, Universitätsklinikum Bonn, Bonn, Deutschland; 3 Zentrum für Blutgerinnungsstörungen und Transfusionsmedizin Bonn, Bonn, Deutschland

**Keywords:** Alkalische Phosphatase, ALP-Genmutationen, Insuffizienzfraktur, Odontohypophosphatasie, Knochenmineralisierung, Alkaline phosphatase, ALP gene mutations, Insufficiency fracture, Odontohypophosphatasia, Bone mineralization

## Abstract

**Hintergrund:**

Die Hypophosphatasie (HPP) ist eine genetische Erkrankung, die durch eine oder mehrere Mutationen im Gen für alkalische Phosphatase (ALP) verursacht wird, verantwortlich für die Kodierung der gewebespezifischen ALP und für den Mineralisierungsprozess.

**Ziel der Arbeit:**

Bestimmung der Prävalenz der HPP bei rheumatologischen Patienten.

**Material und Methoden:**

Retrospektive Analyse der Krankenakten aller erwachsener Patienten mit pathologisch erniedrigten gesamt ALP-Werten (<35 U/l), die zwischen Januar 2017 und Juni 2019 in der Rheumatologie der Medizinischen Klinik III am Universitätsklinikum Bonn behandelt wurden. Die Analyse wurde in Bezug auf klinische Zeichen sowie auf die Ergebnisse der Gentests für HPP untersucht.

**Ergebnisse:**

Bei 60 von 2289 Patienten (2,62 %) zeigten sich pathologisch niedrige ALP-Werte, bei 30 von ihnen (1,31 %) wurden persistierend niedrige ALP-Werte festgestellt. Bei 19 dieser 30 Patienten wurde ein Gentest für ALP-Genmutationen durchgeführt. Sieben der 19 Patienten (36,84 %) hatten HPP-Zeichen (Insuffizienzfrakturen oder schlechter Zahnstatus seit der Kindheit), alle mit pathologischer ALP-Mutation. Drei dieser Patienten (15,78 %) hatten jeweils eine Insuffizienzfraktur mit normwertiger Knochendichtemessung in der Vorgeschichte. Insgesamt 13 von 19 Patienten wiesen (68,42 %) Mutationen im *ALP*-Gen auf. Interessanterweise wurde keine Assoziation mit einer Chondrokalzinose festgestellt.

**Diskussion:**

Die HPP scheint eine unterdiagnostizierte Erkrankung mit einem höheren Anteil betroffener Patienten, welche in der Rheumatologie vorstellig werden, zu sein. Daher sollten zukünftige Studien darauf abzielen, ein Diagnostikprotokoll in der klinischen Praxis zu entwickeln.

## Abstract (en)

**Background:**

Hypophosphatasia (HPP) is a genetic disorder caused by one or more mutations in the alkaline phosphatase (*ALP*) gene, responsible for encoding tissue-specific ALP and for the mineralization process.

**Objective:**

Identification of the prevalence of HPP in rheumatology patients.

**Material and methods:**

Medical records of all adult rheumatology patients with pathologically low total ALP levels (<35 U/L) treated in the Department of Rheumatology at the Clinic of Internal Medicine III, University Hospital Bonn between January 2017 and June 2019, were retrospectively examined for clinical signs as well as for results of genetic tests for HPP.

**Results:**

In 60 out of 2289 patients (2.62%) pathologically low ALP levels were detected. Of these 30 (1.31%) were found to have persistently low ALP levels. Genetic testing for *ALP* gene mutations was performed in 19 of these 30 patients and 7 of the 19 patients (36.84%) had HPP signs (insufficiency fractures, or bad dental status since childhood), all with pathologic *ALP* mutations. Of these patients 3 (15.78%) each had a history of insufficiency fracture with normal bone densitometry. Overall, 13 out of the 19 patients (68.42%) had mutations in the *ALP* gene. Interestingly, no association with chondrocalcinosis was detected in any of the patients.

**Conclusion:**

The HPP seems to be an underdiagnosed disease with a higher proportion of affected rheumatology patients. Therefore, future studies should aim to develop a diagnostic protocol in the clinical practice.

## Hintergrund

Die Hypophosphatasie (HPP) ist eine seltene Erkrankung mit einer geschätzten Prävalenz zwischen 1/100.000 und 1/300.000 für schwere Formen und 1/6370 für mittelschwere Formen in der europäischen Bevölkerung [19]. Bisher liegen jedoch keine epidemiologischen Studien zur Prävalenz der HPP bei rheumatologischen Patienten vor. Die HPP wird durch eine oder mehrere Mutationen im Gen der alkalischen Phosphatase (ALP) verursacht, welches verantwortlich für die Kodierung des TNSALP („tissue-nonspecific alkaline phosphatase protein“) ist. Dieses Enzym ist an der Dephosphorylierung einer Vielzahl von Substraten beteiligt, einschließlich anorganischem Pyrophosphat (PPi), Phosphatidylethanolamin (PEA) und Pyridoxal-5′-phosphat (PLP) [17, 18]. Akkumuliertes extrazelluläres anorganisches Pyrophosphat führt zu einer beeinträchtigten Mineralisierung des Hartgewebes und erklärt somit die klinischen Manifestationen der HPP, zu denen Pseudofrakturen und vorzeitiger Zahnverlust gehören [24]. Bisher wurden insgesamt 388 mit HPP assoziierte Mutationen beschrieben [16]. Es gibt keine direkte Kausalität zwischen Mutationen und klinischen Manifestationen. Eine biallelische Mutation („compound heterozygot oder homozygot“) führt meistens zu einer schwereren Manifestation als eine heterozygote Mutation [7]. Die schwere Form dieser Störung könnte durch autosomal-rezessive Vererbung oder durch einen dominanten negativen Effekt auf eine autosomal-dominante Mutation erklärt werden. Der Grad der Hypophosphatasämie und die TNSALP-Substratakkumulation spiegeln den Schweregrad der HPP wider [25]. Niedrige Konzentrationen der ALP im Serum in Verbindung mit der Anreicherung natürlicher Substrate des TNSALP und klinischen sowie radiologischen Befunden sind typisch für die HPP. Laboruntersuchungen ergeben bei HPP-Patienten in der Regel normale bis erhöhte Kalzium- und Phosphatwerte, auf diese Weise können andere Krankheiten wie Hypophosphatasämie bei Unterernährung oder Rachitis häufig schon ausgeschlossen werden.

Die HPP wurde in 7 klinische Formen eingeteilt, darunter die perinatale, infantile, pädiatrische, erwachsene, odonto-, pseudo- und benigne pränatale HPP. Die klinische Manifestation der HPP kann zwischen schweren (hauptsächlich bei perinatalem und infantilem HPP) bis mittelschweren oder leichten (bei Erwachsenen) Erscheinungsformen variieren [6, 9, 27]. Die HPP bei Erwachsenen wird typischerweise im mittleren Alter entdeckt [10, 13]. Die klinische Manifestation der HPP bei Erwachsenen umfasst den Verlust der Mineralisierung, der zu wiederkehrenden Stressfrakturen (z. B. metatarsal) führt, Rachitis in der Vorgeschichte im Kindesalter sowie Muskel- und/oder Gelenkschmerzen [9, 29]. Diese Hypomineralisierung wird durch die Akkumulation von PPi in der extrazellulären Matrix verursacht, die die Bildung von Hydroxylapatitkristallen hemmt [20]. Diese Hypomineralisierung betrifft auch den azellulären Zement, der die Zahnwurzel bedeckt. Aus diesem Grund ist der vorzeitige Verlust des Milch- oder bleibenden Gebisses eine weitere klinische Manifestation [13]. Darüber hinaus führt der Anstieg der endogenen PPi-Spiegel zu Ablagerungen von Calciumpyrophosphatdihydratkristallen im Gelenkknorpel, die CPPD-Arthropathie (Chondrokalzinose) und Enthesiopathie verursachen und bei erwachsenen HPP-Patienten zu Schmerzen des Bewegungsapparates führen können [1, 9, 15, 26]. Aufgrund der charakteristischen Hypomineralisation wird die HPP häufig fälschlicherweise als Osteoporose diagnostiziert und mit Antiresorptiva wie Bisphosphonaten behandelt. Diese Medikamente verschlechtern die Prognose [22]. Paradoxerweise wurde auch beobachtet, dass die Kristallarthropathie (Chondrokalzinose) und die diffuse idiopathische Skeletthyperostose (DISH) die adulte Form der HPP komplizieren [5, 23, 25]. Aufgrund der Verwechslung der HPP mit vielen anderen Diagnosen (Rachitis, Osteomalazie, Osteoporose) ist die Implementierung eines Protokolls in der klinischen Praxis zur Frühdiagnose der HPP essentiell.

## Ziel der Arbeit

Die Bestimmung der Prävalenz der HPP bei rheumatologischen Patienten.

## Material und Methoden

Die Krankenakten aller erwachsenen Patienten mit pathologisch niedrigen ALP-Werten (Gesamt-ALP < 35 IU/l), die zwischen Januar 2017 und Juni 2019 in der Rheumatologie der Medizinischen Klinik III am Universitätsklinikum Bonn vorstellig wurden, wurden retrospektiv auf klinische Zeichen (Insuffizienzfrakturen, schlechter Zahnstatus seit der Kindheit, Chondrokalzinose) sowie auf Ergebnisse eines HPP-Gentests untersucht. Patienten mit persistierenden ALP-Spiegeln unterhalb des Referenzbereiches (35 IU/l bei 37 °C, ALP2, cobas c702, Roche Diagnostics, Mannheim, Deutschland) wurden identifiziert, eine einzelne ALP-Messung unter der Norm führte zum Ausschluss. Die Patienten mit sekundärer Hypophosphatasämie (meistens durch Hypokalziämie bei Unterernährung, unter Bisphosphonate/Denosumab-Therapie oder seltener bei Hyperparathyreoidismus, Cushing-Syndrom, Hypothyreose, chronischer Diuretikagabe und Alkoholismus) wurden ausgeschlossen [12]. Wir bestimmten das Kalzium und das anorganische Phosphat im Serum. Ebenfalls wurden andere Ursachen einer sekundären Hypophosphatasämie (Eiweißmangel, Folsäure und Vitamin‑B_12_-Mangel) durch einen normwertigen MCV („mean cell volume“) ausgeschlossen. Keiner der Probanden wurde mit einer Chemotherapie behandelt.

**Figure 1:**
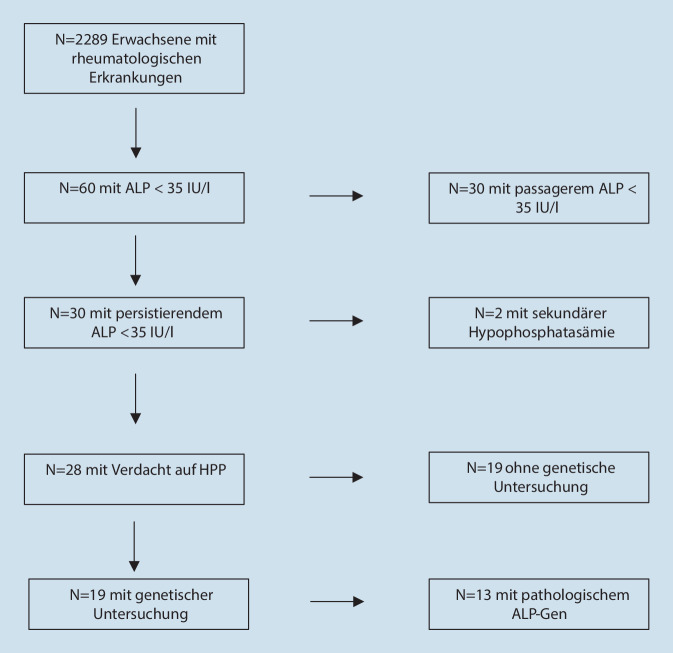

Bei den verbleibenden Patienten mit persistierend niedrigen ALP-Werten < 35 U/l wurden die im Rahmen der klinischen Diagnostik durchgeführten genetischen Untersuchungen retrospektiv ausgewertet (Abb. 1). Zudem wurde die Krankenakte in Papierform sowie im elektronischen Krankenhaussystem ORBIS auf klinische Zeichen einer HPP (Insuffizienzfrakturen, schlechter Zahnstatus seit der Kindheit, Chondrokalzinose) sowie auf die zugrunde liegende Erkrankung untersucht. Wegen der in dieser Studie speziellen rheumatologischen Kohorte litten alle Patienten unter rheumatologischen Symptomen (häufig Arthralgien und Myalgien). Eine genauere Verteilung der klinischen Symptome zwischen HPP und rheumatologischen Erkrankungen war daher nicht möglich.

Bei allen Patienten ist eine aktuelle (<2 Jahre) Knochendichtemessung in Form einer DXA-Messung vorhanden. Für diese Studie wurde eine Verteilung (Tab. 1) zwischen Normbefund (T-Wert > −1), Osteopenie (−1 ≥ T-Wert ≤ −2,5), Osteoporose (T-Wert < −2,5) nach dem T‑Wert definiert.

**Table 1:** 

Alter (in J)	Gender	Osteoporose (T-Wert < −2,5)	Osteopenie (−1 ≥ T-Wert ≤ −2,5)	Osteoporotische Fraktur	Insuffizienzfraktur	Chondrokalzinose	Schlechter Zahnstatus seit Kindheit	Diagnose	ALP (U/l)	Genetisches Test zu Hypophosphatasie-Phänotyp-Zuordnung [11, 21]
80	F	1	0	0	0	0	0	RA/FPA	30	Heterozygote Variante c.455G > A; p. (Arg152his) im Exon 5 des *ALPL*-Gens. Beschrieben bei einem Patienten mit letaler perinataler Hypophosphatasie
39	F	0	0	0	0	0	0	HM-Syndrom	19	Heterozygote Variante c.1001G > A; p.(Gly334Asp) im Exon 10 des *ALPL*-Gens. Beschrieben bei Odontohypophosphatasie
25	F	0	0	0	0	0	0	SLE	29	Kein Nachweis von pathogenen oder potenziell pathogenen Varianten im *ALPL*-Gen
63	F	0	1	0	1 (Mittelfuß)	0	0	PsA	22	Heterozygote Variante c.1172G > A; p.(Arg391His) im Exon 10 des *ALPL*-Gens. Odontohypophosphatasie. Hypophosphatasie mit Beginn im Kindesalter
68	F	0	0	0	0	0	0	RA	30	Kein Nachweis von pathogenen oder potenziell pathogenen Varianten im *ALPL*-Gen
80	F	1	0	1 (LWK 3)	0	0	0	RA	29	Kein Nachweis von pathogenen oder potenziell pathogenen Varianten im *ALPL*-Gen
35	F	0	0	0	1 (Os metarsale D4 links)	0	1	PsA	18	Heterozygote Variante c.1001G > A; p.(Gly334Asp) im Exon 10 des *ALPL*-Gens. Beschrieben bei Odontohypophosphatasie
61	F	1	0	0	0	0	0	FPA	29	Heterozygote Variante c.(787T > C); p.(Tyr263His) im Exon 7 des *ALPL*-Gens. Erniedrigte KDM. Compound-heterozygot mit anderen Varianten bei HPP
47	M	0	1	0	0	0	1	AS	16	Homozygote Variante c.(787T > C); p.(Tyr263His) im Exon 7 des *ALPL*-Gens + heterozygote Variante c.1144G > A; p.(Val382lle). In der gleichen Konstellation bei einem Patienten mit Odontohypophosphatasie
52	F	0	1	0	0	0	0	RA	30	Kein Nachweis von pathogenen oder potenziell pathogenen Varianten im *ALPL*-Gen
39	M	0	1	0	0	0	1	SpA	33	Heterozygote Variante c.406C > T/p.(Arg136Cys) im Exon 5 des *ALPL*-Gens. HPP mit Beginn im Kindesalter sowie Odontohypophosphatasie jeweils compound-heterozygot
70	F	0	0	0	0	0	0	RA	29	Heterozygote Variante c.(787T > C); p.(Tyr263His) im Exon 7 des *ALPL*-Gens. Erniedrigte KDM. Compound-heterozygot mit anderen Varianten bei HPP
53	M	0	0	0	0	0	0	RA	24	Heterozygote Deletion c.1114_1115del/p.(Leu372Aspfs*32) im Exon 10 des *ALPL*-Gens. Nicht in der Literatur beschrieben und wurde in silico als pathogen eingeschätzt!
57	M	0	0	0	0	0	0	IgG4-Erkrankug	31	Kein Nachweis von pathogenen oder potenziell pathogenen Varianten im *ALPL*-Gen
45	F	0	0	0	0	0	0	RA	34	Kein Nachweis von pathogenen oder potenziell pathogenen Varianten im *ALPL*-Gen
65	F	0	1	0	0	0	0	FPA	32	Heterozygote Variante c.211C > A/p.(Arg71Ser) im Exon 4 des *ALPL*-Gens. Hypophosphatasie kindlichen Typs mit Verbesserung in erwachsenem Alter
46	F	0	0	0	0	0	0	PsA	24	Heterozygote Variante c.(455G > A); p.(Arg152His) im Exon 5 undc.(787T > C); p.(Tyr263His) im Exon 7 des *ALPL*-Gens. Das Allel 455A mit letaler perinataler HPP und das 787C mit erniedrigten KDM + mit HPP compound-heterozygot mit anderen Varianten
47	F	0	0	0	0	0	0	Sjögren-Syndrom	34	Heterozygote Variante c.(787T > C); p.(Tyr263His) im Exon 7 des *ALPL*-Gens. Erniedrigte KDM. Compound-heterozygot mit anderen Varianten bei HPP
50	F	0	0	0	1 (Kondylen D4 DIP links)	0	1	RA	11	Heterozygote Variante c.1187T > C, p.Phe396Ser (Variante unklarer Signifikanz-VUS Klasse 3, Variante uneinheitlich bewertet)

*FA* Fingerpolyarthrose, *RA* rheumatoide Arthritis, *PsA* Psoriasisarthritis, *SpA* Spondyloarthritis, *HM* Hypermobilität, *HPP* Hypophosphatasie, *ALP* alkalische Phosphatase

Wegen der Seltenheit der Erkrankung und der sehr variablen klinischen Erscheinungen sind bis jetzt keine definitiven Diagnosekriterien entwickelt worden. In dieser Studie wurde die Diagnose HPP anhand des Vorhandenseins einer erniedrigten ALP mit einem pathologischem ALP-Gentest unabhängig von den klinischen Manifestationen gestellt.

Alle Mutationsanalysen wurden im Zentrum für Blutgerinnungsstörungen und Transfusionsmedizin (CBT, Bonn, Deutschland) durchgeführt. Die genomische DNA wurde aus EDTA-behandeltem Vollblut gemäß den Anweisungen des Herstellers unter Verwendung des genomischen DNA-Reinigungskits von Wizard (Promega, Mannheim, Deutschland) isoliert. Die Kodierungsregionen der Exons 2 bis 12 einschließlich 20 bp flankierender nicht codierender Introngrenzen auf jeder Seite der ALP-Exons (OMIM-G: 171760; NM_000478.5 ALP) und der bekannten regulatorischen pathogenen ALP-Variante c.−195 C > T (c-44655 relativ zum Initiationscodon in der Human Gene Mutation Database auch genannt) wurden durch Touchdown-Polymerasekettenreaktion (PCR) amplifiziert, die zuvor von Korbie und Mattick beschrieben wurde [11]. Die Sequenzanalyse der PCR-Produkte wurde mit der Sanger-Sequenzierungsmethode (ABI 3730xl Analyzer) durchgeführt (Komplettsequenzierung mittels Next-Generation-Sequencing). Die analysierten Sequenzen wurden ausgerichtet und mit der ALP-Referenz verglichen. Alle Sequenzunterschiede zu den Referenzsequenzen (Sequenzvarianten) wurden, basierend auf den Richtlinien für die Interpretation von Sequenzvarianten des American College of Medical Genetics and Genomics (ACMG), interpretiert und in eine von 5 Kategorien von Sequenzvarianten eingeteilt (pathogen [P], wahrscheinlich pathogen [LP], Varianten von unklarer Signifikanz [VUS], wahrscheinlich gutartig [LB] und gutartig [B][21]).

## Ergebnisse

In der Datenbank der Rheumatologie der Medizinischen Klinik III am Universitätsklinikum Bonn wurden zwischen dem 01.01.2017 und dem 30.06.2019 insgesamt 2289 ALP-Messungen durchgeführt. In dieser Kohorte identifizierten wir 60 Patienten (2,62 %) mit ALP-Spiegeln unterhalb des Referenzbereiches (35 IU/l bei 37 °C). Bei 30 Patienten (1,31 %) zeigte sich nur eine einzelne ALP-Messung unter der Norm, was zum Ausschluss führte. Zwei Probanden mit sekundärer Hypophosphatasämie (durch Hypokalziämie bei Unterernährung) wurden ebenfalls ausgeschlossen. Bei den verbleibenden 28 Patienten mit ≥ 2 ALP-Werten < 35 U/l wurden bei 19 Patienten im Rahmen der klinischen Diagnostik genetische Untersuchungen bei Verdacht auf HPP durchgeführt.

### Klinische und biochemische Merkmale der Patienten mit Hypophosphatasämie (Tab. 1)

Bei keinem der 19 Patienten (4 Männer und 15 Frauen) wurde zuvor eine HPP diagnostiziert. Die Hauptdiagnose jedes Patienten ist in Tab. 1 aufgeführt, einschließlich der Diagnosen, welche im Zusammenhang mit einer HPP stehen, wie Arthrose (*n* = 4), periphere Arthritis (RA, PsA, SpA, *n* = 12), Kollagenosen (*n* = 2), Osteoporose (T-Wert < −2,5, *n* = 3), Osteopenie (−1 ≥ T-Wert ≤ −2,5, *n* = 6). Bei keinem wurde in der Kindheit eine HPP oder Rachitis diagnostiziert. Sieben der 19 Patienten (36,84 %) hatten HPP-bezogene klinische Zeichen (Insuffizienzfrakturen, schlechter Zahnstatus seit der Kindheit), 4 dieser 19 Patienten (21,05 %, alle Frauen) hatten nicht osteoporotische Insuffizienzfrakturen (Mittelfuß, Os metarsale D4 links, Kondylen D4 DIP links). Bei keinem der 19 Patienten wurde eine Chondrokalzinose diagnostiziert.

### Pathologische ALP-Genmutationen (Tab. 1)

Bei 13 (4 Männer und 9 Frauen) der 19 Patienten (68 %) zeigten sich pathologische ALP-Genmutationen [21] mit denen in Zusammenhang mit laborchemischen Parametern (ALP < 35 U/l) die Diagnose Hypophosphatasie gestellt wurde. Drei von 4 Patienten mit einer positiven Frakturanamnese, alle mit Insuffizienzfraktur und normwertiger Knochendichtemessung, zeigten einen positiven Gentest (d. h. eine Mutation im *ALP*-Gen). Eine 80-jährige Patientin mit einer LWK-3-Impressionsfraktur und Osteoporose (T-Wert LWK 1–4: −3) unter Zoledronsäure-Therapie zeigte einen negativen ALP-Gentest. Alle 4 der 19 Patienten (21,05 %), die über einen schlechten Zahnstatus seit der Kindheit berichteten, zeigten einen positiven Gentest. Bei den Patienten mit Insuffizienzfrakturen wurde bis dato keine spezielle antiresorptive Therapie durchgeführt.

Bei einer neu aufgetretenen Mutation im *ALP*-Gen, die als pathologisch klassifiziert wurde [21], zeigte sich keine Assoziation mit Frakturen oder schlechtem Zahnstatus seit der Kindheit, während bei einer homozygoten Mutation im *ALP*-Gen, wie in der Literatur vorbeschrieben, eine Assoziation mit einer Odontohypophosphatasie bestehen könnte.

## Diskussion

Unsere Studie zeigt, dass die HPP eine unterdiagnostizierte Erkrankung ist, besonders unter rheumatologischen Patienten. In unserer Kohorte zeigten sich bei 1,31 % der Patienten (ca. 0,12 % in der gesunden spanischen Bevölkerung), die 2017 bis 2019 in der Rheumatologie am Universitätsklinikum Bonn behandelt wurden, anhaltend niedrige ALP-Serumspiegel [8]. Diese Störung wird in der klinischen Praxis häufig übersehen, da niedrige Serum-ALP-Spiegel im Gegensatz zu erhöhten Serum-ALP-Spiegeln häufig nicht die Aufmerksamkeit vieler Ärzte auf sich ziehen.

Ein niedriger ALP-Spiegel im Serum ist nicht gleichbedeutend mit einer HPP. Einige klinische Zustände können auch mit einer Verringerung des zirkulierenden ALP-Spiegels verbunden sein (wie oben in der Methodik referiert wurden), was die Diagnose einer HPP maskieren kann [4, 14].

Unsere Studie ergab bei 13 (4 Männer und 9 Frauen) der 19 rheumatologischen Patienten mit persistierender Hypophosphatasämie eine ALP-Genmutation. In Übereinstimmung mit dem in der Studie von McKiernan et al. [14] angegebenen prozentualen Anteil fanden wir bei 3 Patientinnen laborchemische und klinische Merkmale einer HPP (niedriger ALP-Gesamtserumspiegel und eine Insuffizienzfraktur) (Tab. 1). Darüber hinaus identifizierten wir 10 Patienten (7 Frauen und 3 Männer) mit positivem ALP-Gentest, niedrigem ALP-Spiegel im Serum, jedoch ohne Frakturanamnese. Bei 4 (21 %) dieser 19 Patienten zeigte sich jedoch ein schlechter Zahnstatus seit der Kindheit, aus diesem Grund könnte diese Symptomatik zur Odontohypophosphatasie passen [28]. Im Gegensatz zu anderen Studien litt keiner unserer Patienten unter einer Chondrokalzinose. Zusammenfassend zeigten sich nur bei 7 von 19 Patienten mit erniedrigtem ALP-Wert klinische Zeichen (Insuffizienzfrakturen, schlechter Zahnstatus seit der Kindheit) einer HPP.

Sechs Patienten mit negativem Gentest (5 Frauen und 1 Mann) wiesen pathologisch niedrige ALP-Spiegel auf, zeigten jedoch keine klinischen Manifestationen einer HPP.

Bei einem männlichen Patienten zeigte sich eine neue ALP-Mutation, die in der Literatur als pathologisch klassifiziert wurde. Der klinische Phänotyp dieser neuen Mutation ist nicht spezifisch, da dieser Patient keine Frakturen und keinen schlechten Zahnstatus seit der Kindheit aufwies. Die Schwierigkeit bei der Verknüpfung des Genotyps mit dem Phänotyp kann durch den Einfluss anderer genetischer, epigenetischer oder nichtgenetischer Faktoren bei dieser Störung erklärt werden. Die bioinformatischen pathogenen Prädiktionsinstrumente schlagen vor, dass alle in unserer Studie gefundenen Mutationen einschließlich der neu identifizierten genetischen Variante pathogen sind (gemäß den Klassifizierungskriterien des American College für medizinische Genetik und Genomik [ACMG]) [21].

Ähnlich wie in anderen Studien fanden wir in unserer rheumatologischen Kohorte einen höheren Anteil an Frauen (69 %) als an Männern (31 %), bei denen ein positiver ALP-Gennachweis entdeckt wurde [3, 14]. Es wurden in unserer rheumatologischen Kohorte mit persistierend niedrigen ALP-Serumspiegeln signifikante Geschlechtsunterschiede (15 Frauen, 78 % vs. 4 Männer, 22 %) festgestellt.

Aus diesem Grund können wir ähnlich zu anderen Studien bestätigen, dass die HPP in unserer Studienpopulation bei Frauen häufiger vorkommt als bei Männern. Im Gegenteil zeigte sich in einer größeren spanischen Population kein Geschlechtsunterschied bei Patienten mit anhaltend niedrigen ALP-Serumspiegeln (54 % Frauen, 46 % Männer). Dies liegt am ehesten daran, dass der größte Anteil der rheumatologischen Patienten weiblich ist.

Zusammenfassend lässt sich feststellen, dass die HPP eine unterdiagnostizierte Erkrankung mit einem höheren Anteil an betroffenen rheumatologischen Patienten ist, als bisher angenommen (mindestens 0,56 % der rheumatologischen Patienten vs. 0,01 % in einer spanischen gesunden Bevölkerung) [2]. Nach dem in der vorliegenden Arbeit beschriebenen Protokoll identifizierten wir 13 (0,56 %) betroffene rheumatologische Patienten ohne Vordiagnose einer HPP unter den 19 untersuchten Probanden bei einer Gesamtzahl von 2289 Patienten. Bei 9 von 28 Probanden – also einem Drittel – lag keine genetische Untersuchung vor, daher könnte die Prävalenz möglicherweise unterschätzt worden sein.

Unter den 13 von HPP betroffenen Patienten fanden wir eine neue ALP-Mutation, die zuvor nicht beschrieben wurde. Diese genetische Variante ist mit der klinisch milden Form der Erwachsenen-HPP assoziiert. Zusätzlich fanden wir 6 weitere rheumatologische Patienten mit negativen genetischen Ergebnissen, die erwartungsgemäß keine typischen HPP-Symptome aufwiesen. Aufgrund der klinischen Merkmale könnte die HPP fälschlicherweise mit Osteoporose oder anderen Knochenerkrankungen verwechselt werden, die häufig nicht die Aufmerksamkeit der Ärzte auf sich ziehen. Die Behandlung dieser Patienten mit Antiresorptiva (Bisphosphonate) ist unter rheumatologischen Patienten häufig, was zu einer Verschlechterung ihrer Prognose führen kann. Daher ist es wichtig, ein korrektes klinisches Assessment zu entwickeln, um eine adäquate Diagnose dieser Erkrankung zu ermöglichen. Aus diesem Grund wäre die Bestimmung der ALP bei rheumatologischen Patienten als Standarduntersuchung zu empfehlen, bei persistierend niedrigen Werten und nach Ausschluss sekundärer Ursachen (Kalzium, anorganisches Phosphat, Pyridoxalphosphat, MCV, Bisphosphonate/Denosumab) ist eine genetische Untersuchung sinnvoll. Unsere Studie hat natürlich Schwächen, die dem inhärenten retrospektiven Design originieren. Da die Variablen, die der Studie zugrunde liegen, retrospektiv gewonnen wurden, ist u. a. eine Bestimmung des Pyridoxalphosphat nicht erfolgt. Weitere prospektive Studien sind erforderlich um Frühdiagnoseprotokolle zu entwickeln und zu untersuchen.

## Fazit für die Praxis

- Die Hypophosphatasie ist bei rheumatologischen Patienten häufiger, als man denkt, und kann mit allgemeinen Symptomen wie Arthralgien, Myalgien, Frakturen, schlechtem Zahnstatus seit der Kindheit und Osteoporose vergesellschaftet sein.
- Die Messung der alkalischen Phosphatase ist von hoher Relevanz, eine persistierende Hypophosphatasämie (<35 U/l) kann ein Hinweis für die Erkrankung sein.
- Nach Ausschluss sekundärer Ursachen der Hypophosphatasämie (Hypokalziämie z. B. bei Unterernährung) sollte eine genetische Untersuchung auf Hypophosphatasie angestrebt werden.
